# FGF8 induces epithelial-mesenchymal transition and promotes metastasis in oral squamous cell carcinoma

**DOI:** 10.1038/s41368-021-00111-x

**Published:** 2021-03-01

**Authors:** Yilong Hao, Yanxuan Xiao, Xiaoyu Liao, Shuya Tang, Xiaoyan Xie, Rui Liu, Qianming Chen

**Affiliations:** 1 The Affiliated Hospital of Stomatology, School of Stomatology, Zhejiang University School of Medicine and Key Laboratory of Oral Biomedical Research of Zhejiang Province, Hangzhou, China; 2 State Key Laboratory of Oral Diseases & National Clinical Research Center for Oral Diseases & Chinese Academy of Medical Sciences Research Unit of Oral Carcinogenesis and Management & West China Hospital of Stomatology, Sichuan University, Chengdu, China; 3 Department of Stomatology, The Second Xiangya Hospital, Central South University, Changsha, China

**Keywords:** Oral cancer detection, Cell invasion

## Abstract

Oral squamous cell carcinoma (OSCC) is one of the most common cancers worldwide, and with 354 864 new cases each year. Cancer metastasis, recurrence, and drug resistance are the main causes to cripples and deaths of OSCC patients. As potent growth factors, fibroblast growth factors (FGFs) are frequently susceptible to being hijacked by cancer cells. In this study, we show that FGF8 is upregulated in OSCC tissues and high FGF8 expression is related with a set of clinicopathologic parameters, including age, drinking, and survival time. FGF8 treatment enhances the invasive capability of OSCC cells. Lentivirus-based FGF8 expression promotes OSCC metastasis in a mouse lung metastasis model. Further, mechanistic study demonstrates that FGF8 induces epithelial–mesenchymal transition (EMT) in OSCC cells. These results highlight a pro-metastatic function of FGF8, and underscore the role of FGF8 in OSCC development.

## Introduction

According to the global cancer statistics 2018, lip and oral cavity cancers are one of the most common cancers globally, and approximately 354 864 new cases and 177 384 cancer-related deaths are reported^1^. Over 90% of oral cancers are diagnosed as oral squamous cell carcinomas (OSCC)^2^. Many systematic therapeutic strategies have been applied in OSCC treatment^3^, however, the overall 5-year survival rate is still less than 60%^4–7^, and metastasis has been associated with a poor prognosis. Lymph node metastasis is frequently detected in OSCC patients, and is found to be associated with clinicopathological parameters, such as tumor volume and histologic differentiation^8^. Although the incidence of distant metastasis is rare compared to other cancers, its occurrence is determinant to patient prognosis and clinical outcome^9–11^. However, OSCC metastasis is a multiple and complex process^4,12–14^, and the key oncogenic factors involved in this process are not fully illustrated. Therefore, a better understanding of the mechanisms underlying OSCC metastasis is still needed.

There are 22 mammalian fibroblast growth factors (FGFs), which can be subdivided into six subfamilies based on protein sequence homology and phylogeny^15,16^. FGFs can act as morphogens, mitogens, and inducers of angiogenesis, when FGFs bind and activate FGF receptors (FGFRs), leading to activation of a series of biological processes^16–22^. FGFs are frequently upregulated in invasive tumors, making FGF signaling susceptible to be hijacked by cancer cells, facilitating tumor metastasis^15,18–20^. It is reported that FGF1, FGF7, and FGF10 can induce epithelial-mesenchymal transition (EMT) in bladder carcinoma cells^18^. FGF1, FGF2, FGF6, FGF9, and FGF17 are shown to be overexpressed in prostate cancer^18,19,23^. FGF8, FGF9, FGF10, FGF18, and FGF23 are involved in the progression of colorectal cancer, and FGF9 expression is negatively correlated with patients’ survival^19,21,24^.

FGF8 is expressed in oral and maxillofacial tissues during embryonic development, and regulates EMT and mesenchymal–epithelial transition to facilitate organ formation. FGF8 expression disorder can lead to a variety of oral and maxillofacial developmental defects. In adults, FGF8 is associated with diverse physiologic processes, including angiogenesis, wound repairing, homeostasis, cell differentiation, and cell migration^25^. By contrast, FGF8 is rarely detected in normal adult tissues. However, aberrantly increased FGF8 expression is involved in the development of several forms of hormone dependent cancers, and engineered overexpression of FGF8 is found to promote cancer cell invasion in animal models^20,21,26^. FGF8 can enhance the invasion and migration of prostate cancer cells and promote bone metastasis^26–28^. In a previous study, we reported that LRP6 promoted the expression of FGF8 in OSCC cells, and activation of LRP6 contributed to metastasis and poor prognosis in patients with OSCC. More importantly, in contrast to LRP6 expression alone, the concurrent expression of LRP6 and FGF8 could act as a better factor to predict OSCC patient prognosis^12^. However, the function of FGF8 alone in OSCC metastasis remains unclear.

In this study, FGF8 is found to be highly expressed in OSCC tissues, and is linked with an index of histopathological parameters. Further, we demonstrate that FGF8 treatment promotes EMT and induces an invasive phenotype in OSCC cells.

## Results

### FGF8 is overexpressed in OSCC

**Fig. 1:**
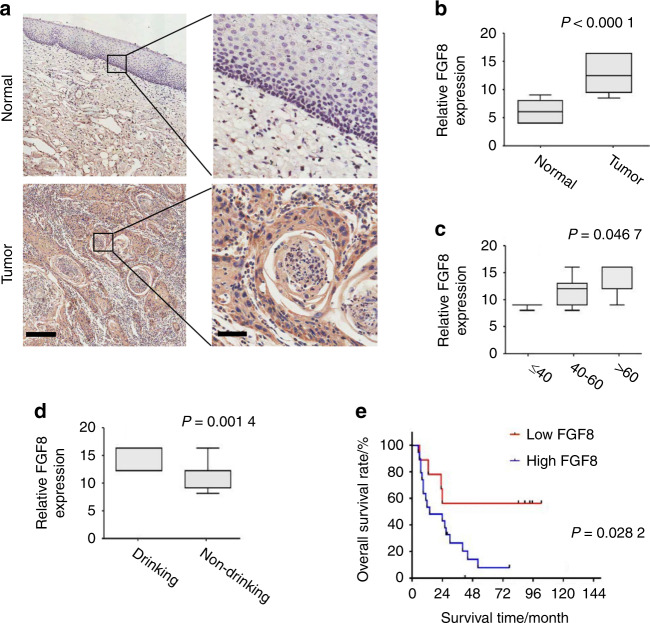
FGF8 is overexpressed in OSCC. **a** Representative images of FGF8 immunostaining using OSCC tissues and normal oral mucosal tissues. Scale bar, left panels, 200 μm; right panels, 50 μm. **b** FGF8 immunostaining intensity in OSCC tissues and normal oral mucosal tissues were analyzed. **c** FGF8 immunostaining intensity in OSCC patients among the different age groups were analyzed. **d** FGF8 immunostaining intensity in OSCC patients with or without drinking were analyzed. **e** Overall survival time of OSCC patients with high or low FGF8 expression was analyzed by Kaplan–Meier analysis

To investigate the potential clinical roles of FGF8 in OSCC, immunohistochemistry staining was performed on a panel of 30 OSCC specimens and 28 adjacent normal oral mucosa specimens. As shown in Fig. 1a, FGF8 signal was positively detected in both the cytoplasm and membrane in tumor cells, whereas only weak staining of FGF8 was observed in majority of normal tissues (*t*-test; OSCC *n* = 30, normal *n* = 28; *P* < 0.000 1; Fig. 1b).

Next, we evaluated the relevance between FGF8 expression and a series of clinicopathologic factors of OSCC patients. FGF8 immunoreactivity was more intense in tumor of elderly patients (one-way ANOVA; ≤40 *n* = 3, 40–60 *n* = 11, >60 *n* = 16; *P* = 0.046 7; Fig. 1c). Further, the level of FGF8 expression was positively associated with drinking (*t*-test; drinking, *n* = 14; non-drinking, *n* = 16; *P* = 0.001 4; Fig. 1d).

In a univariate analysis examining clinic-pathologic prognostic variables, the expression of FGF8 was significantly correlated with overall survival. A Kaplan–Meier survival analysis showed that subjects with high FGF8 expression had a significantly shorter 5-year overall survival time, compared to those subjects with low FGF8 expression (log-rank test, *P* = 0.002 82; Fig. 1e). These results show that FGF8 is highly expressed in OSCC and may act as a potential prognostic marker for predicting patient outcome.

### Analyses of FGF8-associated proteins

**Fig. 2:**
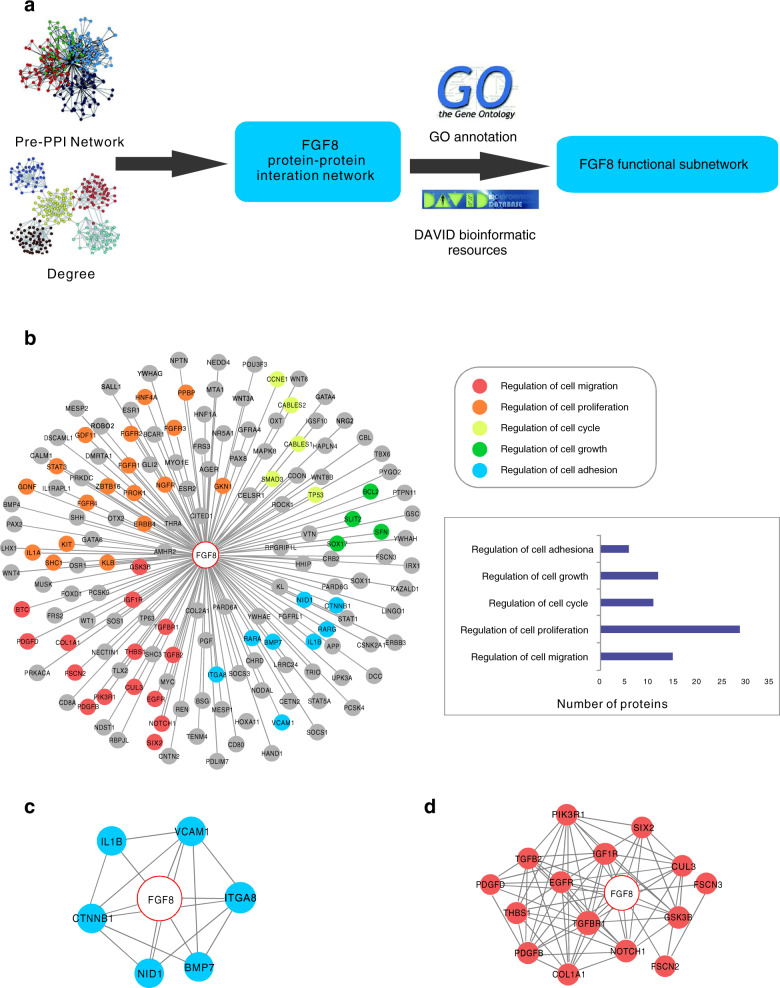
Analyses of FGF8-associated proteins. **a** The general workflow of bioinformatics analysis. **b** Sub-PPI network for FGF8-associated proteins are shown and the predicted FGF8-associated proteins were divided into several groups based on their function. **c** The predicted FGF8-associated proteins involved in cell adhesion are shown. **d** The predicted FGF8-associated proteins involved in cell migration are shown

To explore the tumor-related function of FGF8, bioinformatic analyses was performed to screen the FGF8-related proteins (Fig. 2a). As a result, a total of 158 related proteins were extracted from Pre-PPI network and identified as FGF8-associated proteins. Next, we used the protein-functional GO annotation in the Kyoto encyclopedia of genes and genomes (KEGG) database to perform functional classification and signal pathway analysis of the associated proteins (Fig. 2b). Notably, two clusters of proteins, functioning in cell adhesion or migration, respectively, were found (Fig. 2c, d). These results suggest that FGF8 is likely involved in regulating OSCC metastasis.

### FGF8 promotes OSCC cell invasion and migration

**Fig. 3:**
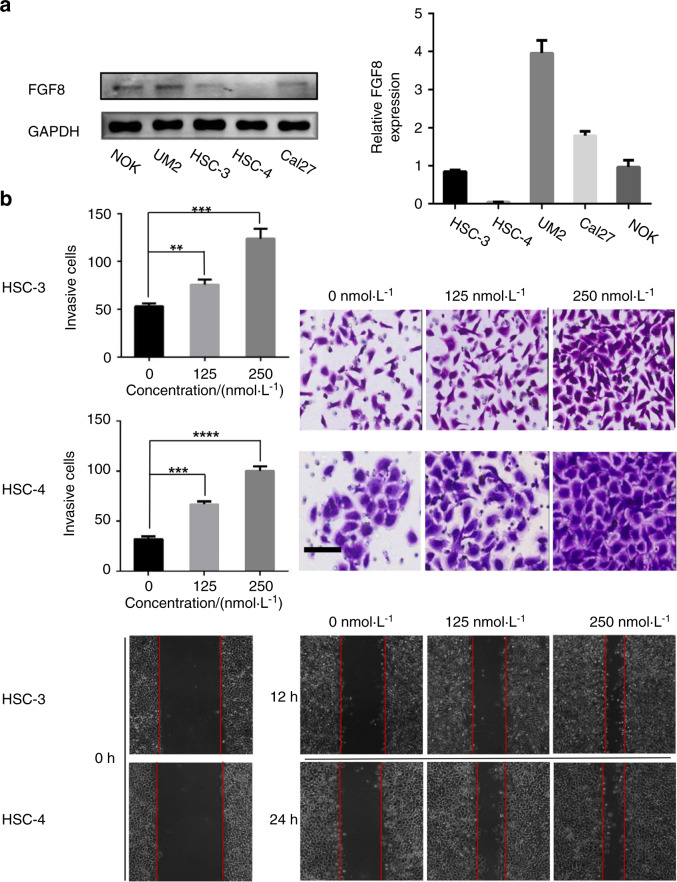
FGF8 promotes OSCC cell invasion and migration. **a** Expression of FGF8 in NOK and several OSCC cell lines was examined by qRT-PCR and immunoblot. **b** HSC-3 or HSC-4 cells were incubated with active FGF8 recombinant protein at indicated concentrations. Cell migration was examined by wound healing assay (upper two panels) and cell invasion was examined by matrigel invasion assay (bottom two panels). **P < 0.01; ***P < 0.001; ****P < 0.000 1. Scale bar, 100 μm

It has been demonstrated that FGF8 is involved in regulating migration and invasion in cancer cells^19,21,24^. As a pilot test, FGF8 expressions in one normal oral keratinocytes (NOK) and four human OSCC cell lines (HSC-4, HSC-3, Cal-27, and UM2) were examined. As shown in Fig. 3a, FGF8 was less expressed in HSC-3 and HSC-4 cell lines at both RNA and protein levels. Therefore, HSC-3 and HSC-4 cell lines were selected as in vitro cell models.

The migratory and invasive capacities of OSCC cells were compared under FGF8 treatment at different concentrations. As shown in Fig. 3b, FGF8 treatment promoted HSC-3 cells migration and increased the invasion potential, as demonstrated by wound healing assay and matrigel invasion assay. Similar results were observed in HSC-4 cells, therefore, such pro-migration and pro-invasion effects of FGF8 were not cell line-specific. Consistently, knockdown of FGF8 impeded the migratory and invasive capabilities of UM2 cells (Fig. S1a, S1b). These results demonstrate that FGF8 promotes migration and invasion in a dose-dependent manner in OSCC cells.

### FGF8 increases OSCC tumor metastasis in mice

**Fig. 4:**
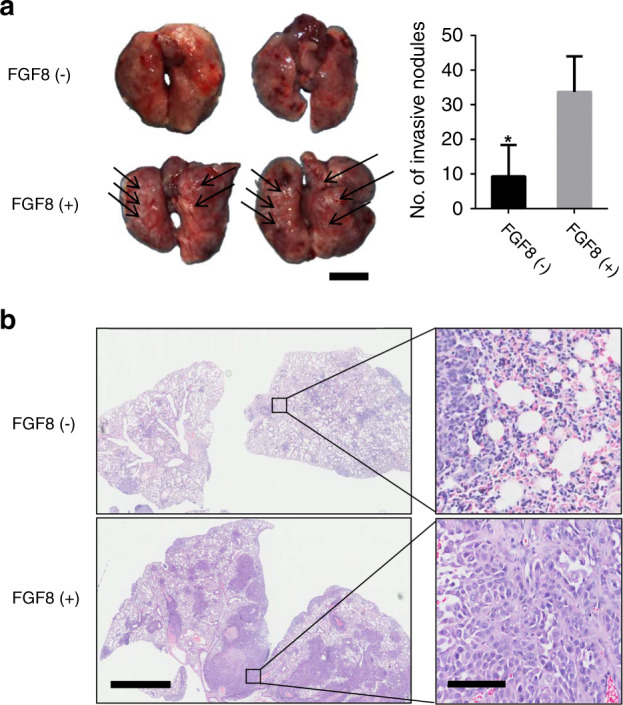
FGF8 increases OSCC tumor metastasis in mice. **a** Representative images of lung metastasis and number of metastases are shown. The number of metastatic nodules are counted. *P < 0.05. Scale bar: 5 mm. **b** H&E staining of the lung tissues. Scale bar: left panels, 2 mm; right panels, 100 μm

To study the effect of FGF8 on tumor metastasis in vivo, FGF8 expression was induced in HSC-3 cells by a lentivirus-based system. FGF8-expressed or mock vector-expressed HSC-3 cells were intravenously injected into the nude mice to establish lung metastasis. The average number of metastatic nodules derived from FGF8-expressed HSC-3 cells was 2.2-fold greater than control cells (*P* < 0.05; Fig. 4a). In addition, the lung metastasis areas formed by FGF8-expressed HSC-3 cells were markedly larger than that formed by control cells, as determined by H&E staining (Fig. 4b). These results show that FGF8 has a positive impact on the OSCC cell metastasis in mice model.

### FGF8 promotes EMT in OSCC cells

**Fig. 5:**
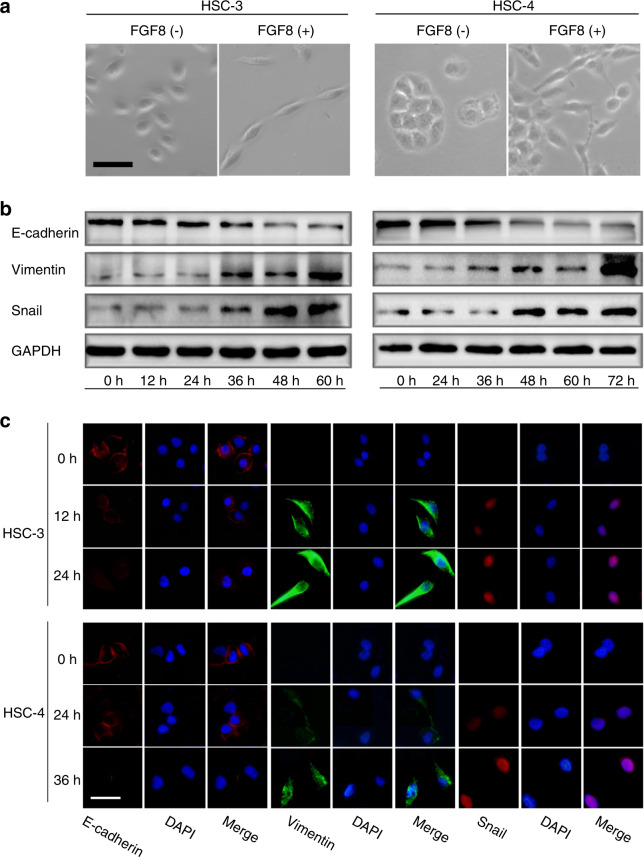
FGF8 promotes EMT in OSCC cells. **a** Representative phase-contrast images of cell morphology of HSC-3 and HSC-4 cells. Scale bar, 20 μm. **b**, **c** Expression of Snail, E-cadherin, and Vimentin was examined by immunoblot (**b**) and immunofluorescent staining (**c**). Scale bar, 30 μm

EMT was involved in the initial steps during cancer metastasis^27^. Therefore, it was our particular interest to examine whether FGF8 plays a role in regulating OSCC cells EMT. As shown in Fig. 5a, FGF8 treatment induced morphological changes in OSCC cells. HSC-3 and HSC-4 cells, which were both sub-rotund or sub-rectangular, changed into a spindle-like shape.

Furthermore, FGF8 treatment also reduced the expression of the epithelial marker E-cadherin, and increased the levels of mesenchymal markers Vimentin and Snail in HSC-3 and HSC-4 cells. These results suggest FGF8 induces a malignant phenotype by promoting EMT in OSCC cells in a time-dependent manner (Fig. 5b, c).

## Discussion

Since cancer metastasis, neoplasm recurrence, and drug resistance are frequently observed in OSCC patients, OSCC is among the most common oral malignancies worldwide, with the 5-year overall survival rate less than 60%. Development of OSCC also causes oral-facial disfigurement and functional defects in chewing, speaking, and swallowing, which largely compromises life quality^12,14,29–32^.

FGF/FGFR signaling is involved in multiple processes during embryonic development and adult homeostasis by regulating cell commitment, differentiation, proliferation, and apoptosis of various types of cells^16^. Increasing evidence indicates that aberrant FGF signaling is frequently observed in various tumors. FGF/FGFR system has important roles in tumor growth, metastasis, and resistance to anticancer therapies^16,17^. Of note, accumulating studies have underlined the role of the transduction network triggered by the aberrant FGF signaling towards a stimulatory interaction between tumor and stromal cells.

In this context, our findings further support the recent discoveries regarding the roles of FGF8 in OSCC. By immunostaining using clinical samples, we show the upregulation of FGF8 expression in OSCC tissues compared to normal tissues. The results also show that FGF8 expression is strongly associated with the habit of drinking. Drinking is considered as one of important risk factors during tumor development. Long-term alcohol abuse can cause salivary gland atrophy and lesions. Therefore, the mucosal surface is directly exposed to carcinogens, thereby increasing the risk of OSCC^33–35^. Notably, our results also show that FGF8 expression is negatively correlated with the survival time of patients, suggesting that FGF8 may be a potential indicator for OSCC prognosis.

EMT is identified as part of the process of invasion and metastasis^36^. EMT can be characterized by changes in cell shape, through which epithelial cells become detached from each other, penetrate the basilar membrane and transform into mesenchymal-like cells with a more flexible and migratory phenotype^37–40^. EMT can be induced by a variety of growth factors, including FGFs. FGFRs are activated after binding to cognate FGFs, and in turn trigger intracellular downstream signaling cascades via phosphorylating the tyrosine residues in their substrates. Our results show a pivotal role of FGF8 in EMT induction in OSCC cell lines. Here, the downregulation of the epithelial marker, E-cadherin, and the upregulation of mesenchymal markers, Vimentin, and Snail, are detected. FGF8 regulates OSCC metastasis probably through inducing EMT. Further work is still needed to identify the intracellular effector proteins that promote EMT under FGF8 treatment.

Though the incidence of distant metastasis is relatively low for OSCC compared with other types of tumor, such as lung or breast cancer, but it remains a crucial determinant for patient prognosis and clinical management. The most common metastatic site is the lung, which accounts for approximately 70% of cases, followed by bone and liver^9–11^. If the process of tumor metastasis can be delayed or impeded, the survival time of patients with advanced cancer will be largely improved^14^. Considering that lymphatic metastasis is more frequent in OSCC patients^41,42^, further study should be conducted to verify the prometastatic role of FGF8 in a lymphatic metastatic model.

In summary, our studies provide evidences regarding the pro-metastatic role of FGF8 in OSCC cells. We also demonstrate aberrant upregulation FGF8 in OSCC, which is associated with the habit of drinking, and patient survival time. This study highlights the role of FGF8 in OSCC development, and will assist the OSCC management.

## Materials and methods

### Clinical samples

Thirty OSCC specimens containing adjacent noncancerous areas and 28 normal oral mucosal tissues for immunohistochemical (IHC) analysis were collected from the Department of Oral and Maxillofacial Surgery, Hospital of Stomatology, Sichuan University. Demographic data and other variables, including dates of diagnoses, site and size of primary tumor, local regional recurrence, and distant metastasis were retrieved from the database provided by the oncology registry. The cancerous or noncancerous areas were identified by two pathologists independently, according to the IHC staining. The pathologists were blinded to patient clinical information. If the evaluations were controversial, the samples were re-evaluated and classified based on the assessment given most frequently by the pathologists. All the samples were obtained with patient’s informed consent. The protocol of the study was approved by the Institutional Ethics Committee of West China Center, Sichuan University, China.

### Immunohistochemistry

Immunohistochemistry Anti-FGF8 rabbit monoclonal antibody (ab81384, 1:200) was purchased from Abcam (Cambridge, MA, USA). Immunohistochemistry was detected on a slide carrying 4-mm-thick tissue from paraffin-embedded tumor species. After baked in a 37 °C oven overnight, all slides were dewaxed in xylene and then rehydrated in ascending series of ethanol. Antigen retrieval was conducted by citrate antigen retrieval solution in an autoclave for 5 min. Three percent of hydrogen peroxide was incubated for 15 min, and normal goat serum working fluid incubated for 15 min at 37 °C after washing for 5 min twice. Then, the sections were exposed to the primary antibodies at 4 °C in the wet box for one night. The slide tissues were washed in PBS for 5 min three times and incubated secondary antibody for 15 min at 37 °C. DAB chromogenic reagents were used to detect the reaction of antigen and antibody and the slides were counterstained in hematoxylin, dehydrated in gradient alcohol, cleared in xylene.

To estimate the score of each section, eight individual fields were chosen by two dependent observers, and 100 cancer cells were counted for each field. We quantitatively scored the tissue sections according to the percentage of positively stained cells and staining intensity as described previously^43^, with minor modifications. We assigned the following proportion scores: 0 if 0% of the tumor cells with positive staining, 1 if 0–10%, 2 if 11%–30%, 3 if 31%–70%, and 4 if 71%–100%. We also rated the intensity of staining on a scale of 0 to 3: 0, negative; 1, weak; 2, moderate; 3, strong and 4, very strong. We then multiplied the proportion score by the intensity score to obtain a total score (range: 0–16). Scores were compared with overall survival duration, which was defined as the time from the date of diagnosis to death or the last known date of follow-up.

### Bioinformatics analysis

Bioinformatics analysis of FGF8-assocaited proteins were performed following previous reports^44^. The protein–protein interaction (PPI) network was conducted based on the identified proteins, and biological evidence was collected from PrePPI to obtain the correlation of protein localization, the correlation of expression, the mutual binding, the upstream-related and downstream-related proteins. Identified FGF8-associated proteins were classified according to the GO (Gene Ontology) Annotation clustering. The network group analysis was conducted via DAVID database (http://david.abcc.ncifcrf.gov/)^45^.

### Cell culture

The HSC-3 and HSC-4 cell lines were provided by State Key Laboratory of Oral Diseases & National Clinical Research Center for Oral Diseases, West China Hospital of Stomatology, Sichuan University. Cells were maintained in Dulbecco’s Modified Eagle’s Medium (DMEM, Gibco, USA) containing 10% fetal bovine serum (Hyclone, USA), penicillin (10^7^ U·L^−1^) and streptomycin (10 mg·L^−1^) at 37 °C in a humidified chamber containing 5% CO_2_. FGF8 siRNAs were purchased from Santa Cruz.

### qPCR

Total RNA of OSCC cell lines was isolated by TRIzol reagent (Invitrogen) and reverse transcript to cDNA with 1 μg RNA in a volume of 20 μL by ExScript TM reagent kit (TaKaRa, Dalian, China) according to the manufacturer’s instructions. The primers detailed sequences were as follows: FGF8: Forward primer: 5′-CGC AAA GCT CAT TGT GGA GA-3′, Reverser primer: 5′-ACA CGC AGT CCT TGC CTT TG-3′; GAPDH: Forward primer: 5′-GAG TCA ACG GAT TTG GTC GT-3′, Reverser primer: 5′-TTG ATT TTG GAG GGA TCT CG-3′. Gene expression level was assessed by SYBR green qPCR SuperMix (Applied Biosystems Life Technologies, Foster, CA) and GAPDH served as an internal reference. The fold-change in the expression of each target mRNA relative to GAPDH was calculated using the CT (2^−ΔΔCT^) method. Each experiment was conducted in triplicate.

### Wound healing assay

When the cells cultured in 6-well plates reached approximately 100%, the wells were gently scratched with a 100 μL pipette tip to create a uniform linear scratch. Then the cells were cultured in serum-free culture medium, and observed and photographed at 0, 12, and 36 h. Cell migration was assessed by percent of wound closure through using Image-Pro Plus Analysis software (Media Cybernetics company, Rockville, MD). All experiments were conducted for three times to obtain the average value.

### Transwell invasion assay

Invasion assays were carried out using 24-well culture plates containing the transwell chamber covered with Matrigel (1:4, BD, USA). 1 × 10^5^ HSC-3 and 2 × 10^5^ HSC-4 cells suspended in serum-free medium were placed in the upper chamber. Five hundred microliter of medium containing 10% FBS were placed in the lower chamber. Cells remaining on the upper chamber were removed using a cotton swab after being incubated at 37 °C for 12–36 h, while cells traversed to reverse face of the membrane were fixed in 4% paraformaldehyde, stained with 1% Crystal Violet, washed three times with PBS, then air dried. The chamber was inverted on a microslide and observed under a microscope. Five fields per chamber were randomly selected for counting the number of invasive cells, and images were taken. Each experiment was conducted for three times.

### In vivo tumor metastasis

All animals were humanely treated under the guidelines of the Institutional Animal Care and Treatment Committee of Sichuan University. 5 × 10^6^ OSCC-FGF8 or OSCC-mock cells were injected into female athymic nude mice (ten mice per group) through the tail vein. Animals were sacrificed 28 days after injection. The lungs were excised and fixed in formalin for standard hematoxylin and eosin (H&E) staining.

### Western blotting

After FGF8 treatment, total proteins of OSCC cells were extracted in RIPA buffer (50 mmol·L^−^^1^ Tris base, 1.0 mmol·L^−1^ EDTA, 150 mmol·L^−^^1^ NaCl, 0.1% SDS, 1% Triton X-100, 1% sodium deoxycholate, and 1% cocktail) and quantified by coomassie brilliant G-250 (Bio-Rad). Samples were separated on 12% or 15% SDS-PAGE and then transferred to PVDF membranes. The membranes were blocked with 5% skim milk in TBST for 1 h at 37 °C and probed with primary antibody overnight at 4 °C. After washing with TBST membranes were incubated with secondary antibody (1:5 000 dilution; Santa Cruz Biotechnology) conjugated to horseradish peroxidase for 1 h at 37 °C. Finally, the proteins were detected by electro-chemiluminescence (ECL) Western blotting reagents. The following primary antibodies were used according to the manufacturer’s instructions: anti-E-cadherin mouse monoclonal antibody (ab1416, 1:1 000), anti-Vimentin rabbit polyclonal antibody (ab137321, 1:1 000), Anti-Snail rabbit polyclonal antibody (ab82846, 1:800), and anti-GAPDH (ab8245, 1:1 000, Abcam).

### Immunofluorescence staining

The OSCC cells were cultured in 24-well cell culture plates, fixed for 15 min with 4% paraformaldehyde and permeabilized with 0.2% Triton X-100 for 20 min. After blocking with normal goat serum working fluid for 1 h at 37 °C, primary antibody was incubated overnight at 4 °C, and then staining was detected with fluorescein-conjugated secondary antibodies (PeproTech; 1:200) for 1 h in dark condition. Finally, cells were stained with 4,6-diamidino-2-phenylindole (DAPI; blue) to show the nuclear position for 5 min. Immunofluorescence signals were examined using a fluorescence microscope (Leica, Bensheim, Germany).

### Lentiviral transduction

Expression of FGF8 were established using a pCDH Lentivector Expression System (System Biosciences, Mountain View, CA) according to the manufacturer’s instructions. Briefly, the shRNAs or cDNAs used in this study were cloned into pCDH lentiviral vector. Lentiviruses were produced by co-transfecting 293T cells with one of the expression plasmids and three packaging plasmids (pLP1, pLP2, and pLP/VSVG). Infectious lentiviruses were harvested 72 h after transfection, centrifuged to remove cell debris, and filtered through 0.45 µm filter (Millipore, Bedford, MA).

## Supplementary information

Supplementary Figure Legends

Figure S1
