# Supplementary material for: FGF8 induces epithelial-mesenchymal transition and promotes metastasis in oral squamous cell carcinoma
Source: Int J Oral Sci. 2021 Mar 1;13:6. doi: 10.1038/s41368-021-00111-x (PMC7921665; doi:10.1038/s41368-021-00111-x)
Supplement: Supplementary file 1 — Supplementary Figure Legends [file 41368_2021_111_MOESM1_ESM.docx]

**Supplementary Figure Legends**

**Figure. S1 FGF8 promotes OSCC tumor invasion and migration.**

(a) UM2 cells were transfected with non-specific siRNA or FGF8 siRNA, and expression of FGF8 mRNA was examined by RT-PCR 48 h after transfection. (b) The migratory and invasive capabilities of UM2 cells were measured by wound healing assay (upper panels) and Matrigel assay (bottom panels).
