# Supplementary figures and images for: FGF8 induces epithelial-mesenchymal transition and promotes metastasis in oral squamous cell carcinoma
Source: Int J Oral Sci. 2021 Mar 1;13:6. doi: 10.1038/s41368-021-00111-x (PMC7921665; doi:10.1038/s41368-021-00111-x)

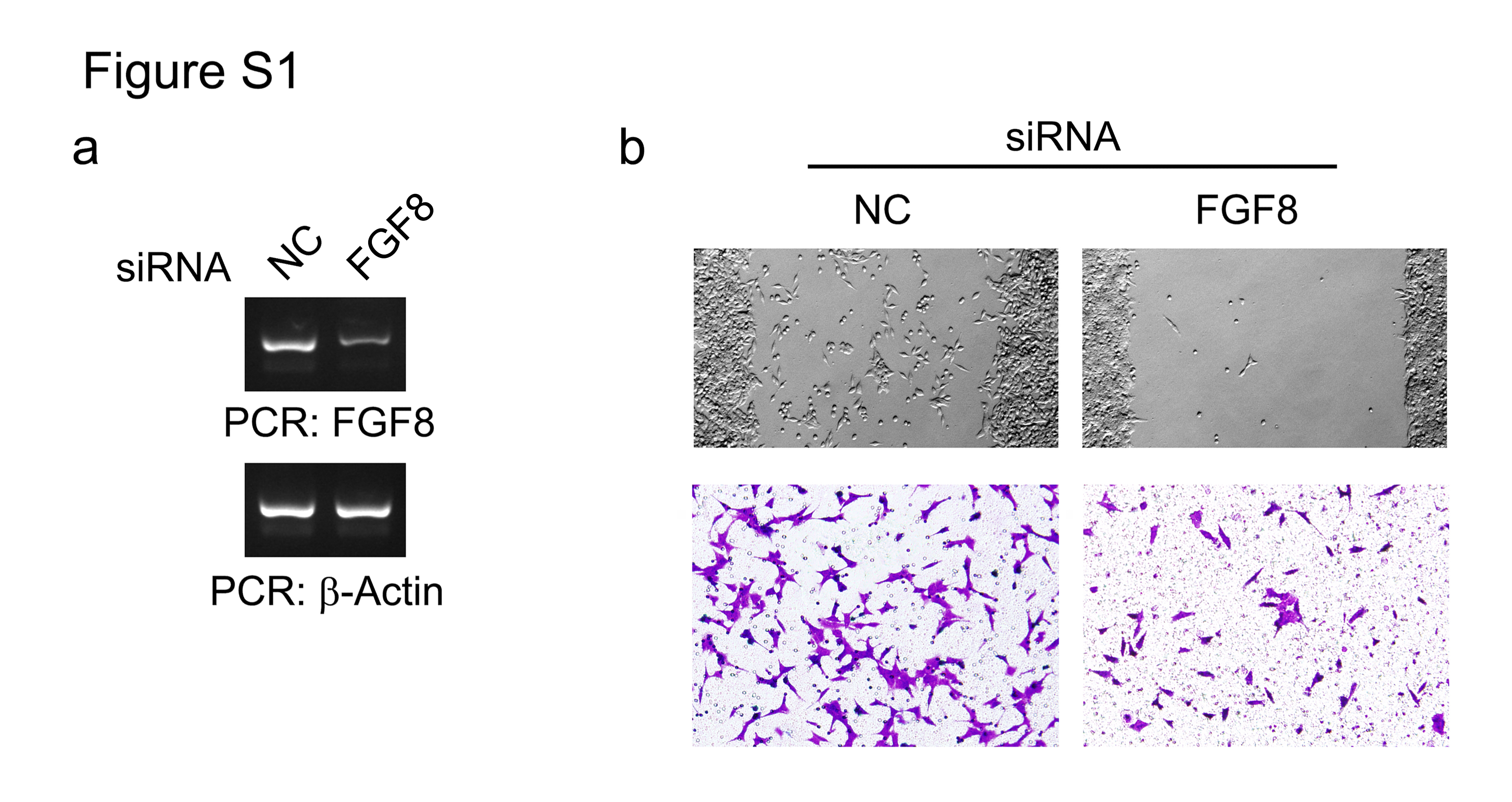

Supplement: Supplementary file 2 — Figure S1 [file 41368_2021_111_MOESM2_ESM.tif]
